# Supplementary material for: The HoxD cluster is a dynamic and resilient TAD boundary controlling the segregation of antagonistic regulatory landscapes
Source: Genes Dev. 2017 Nov 15;31(22):2264–81. doi: 10.1101/gad.307769.117 (PMC5769770; doi:10.1101/gad.307769.117)
Supplement: Supplemental Material [file supp_31_22_2264__index.html]

The HoxD cluster is a dynamic and resilient TAD boundary controlling the segregation of antagonistic regulatory landscapes — Supplemental Material 

# The *HoxD* cluster is a dynamic and resilient TAD boundary controlling the segregation of antagonistic regulatory landscapes

## Supplemental Material

- Supplemental\_Material.pdf
