# Supplementary material for: The HoxD cluster is a dynamic and resilient TAD boundary controlling the segregation of antagonistic regulatory landscapes
Source: Genes Dev. 2017 Nov 15;31(22):2264–81. doi: 10.1101/gad.307769.117 (PMC5769770; doi:10.1101/gad.307769.117)
Supplement: Supplemental Material [file supp_gad.307769.117_Supplemental_Material.pdf]

THE HOXD CLUSTER IS A DYNAMIC AND RESILIENT TAD BOUNDARY CONTROLLING THE SEGREGATION OF ANTAGONISTIC REGULATORY LANDSCAPES

Eddie Rodríguez-Carballo[1], Lucille Lopez-Delisle[2], Ye Zhan[3], Pierre J. Fabre[2#], Leonardo Beccari[1], Imane El-Idrissi[1], Thi Hanh Nguyen Huynh[1], Hakan Ozadam[3], Job Dekker[3] and Denis Duboule[1, 2, 4].

SUPPLEMENTAL MATERIAL

Supplemental Tables 1 to 3

Supplemental Figures S1 to S7

Supplemental Tables S1 to S3

Rodriguez-Carballo307769_Table S1

**Island-2**	**iF**	AATGATACGGCGACCACCGAACACTCTTTCCCTACACGACGCTCTTCCGATCTNNNNGCATTCATCAAGCTGTGATTAGCA
	**iR**	CAAGCAGAAGACGGCATACGAAATCCCATAATATGTAGACTGTAGTGTTGC
**Island-4**	**iF**	AATGATACGGCGACCACCGAACACTCTTTCCCTACACGACGCTCTTCCGATCTNNNNTACAGCCTAGTCTTTTCTCATCACAT
	**iR**	CAAGCAGAAGACGGCATACGATGTAATTATTTCAGGGTTGGAGTAGAATCA
Evx2	**iF**	AATGATACGGCGACCACCGAACACTCTTTCCCTACACGACGCTCTTCCGATCTNNNNTTGGAAACCCTGTGAGCCTAC
	**iR**	CAAGCAGAAGACGGCATACGAGGGAAGAAACCTACCACGACAC
Hoxd13	**iF**	AATGATACGGCGACCACCGAACACTCTTTCCCTACACGACGCTCTTCCGATCTNNNNAAAATCCTAGACCTGGTCATG
	**iR**	CAAGCAGAAGACGGCATACGAGGCCGATGGTGCTGTATAGG
Hoxd4	**iF**	AATGATACGGCGACCACCGAACACTCTTTCCCTACACGACGCTCTTCCGATCTNNNNCAGGACAATAAAGCATCCATAGGCG
	**iR**	CAAGCAGAAGACGGCATACGATCCAGTGGAATTGGGTGGGAT
**LacZ**	**iF**	AATGATACGGCGACCACCGAACACTCTTTCCCTACACGACGCTCTTCCGATCTNNNNTAGTGCAACCGAACGCGAC
	**iR**	CAAGCAGAAGACGGCATACGAGGCAAAGACCAGACCGTTCATAC
**CS38**	**iF**	AATGATACGGCGACCACCGAACACTCTTTCCCTACACGACGCTCTTCCGATCTNNNNTTCCAAGGAGAAAGGTGTTGGTC
	**iR**	CAAGCAGAAGACGGCATACGACAGGGCGTTGGGTCACTCT
**CS65**	**iF**	AATGATACGGCGACCACCGAACACTCTTTCCCTACACGACGCTCTTCCGATCTNNNNTCTAGTGAGCCCCTACCAGGA
	**iR**	CAAGCAGAAGACGGCATACGAGGAGCCTTTGGGGTACACG

Supplemental Table S1. **List of the viewpoints used for 4C-seq**. Sequences of the primers used to generate the 4C libraries. Custom barcodes (4bp) were introduced in between the Illumina adapter sequences and the specific viewpoint sequence in order to multiplex different samples of the same viewpoint.

Rodriguez-Carballo307769_Table S2

Experiment	Viewpoint	Allele	Tissue	Experimental Replicates
4C-seq	Island-2	*Wt*	Distal Forelimb	1
4C-seq	Island-2	*Wt*	Proximal Forelimb	1
4C-seq	Island-2	*del(attP-Rel5)d9lac*	Distal Forelimb	1
4C-seq	Island-2	*del(attP-Rel5)d9lac*	Proximal Forelimb	1
4C-seq	Island-4	*Wt*	Distal Forelimb	5
4C-seq	Island-4	*Wt*	Proximal Forelimb	5
4C-seq	Island-4	*del(10-12)*	Distal Forelimb	1
4C-seq	Island-4	*del(10-12)*	Proximal Forelimb	1
4C-seq	Island-4	*del(9-12)*	Distal Forelimb	1
4C-seq	Island-4	*del(9-12)*	Proximal Forelimb	1
4C-seq	Island-4	*del(1-10)*	Distal Forelimb	1
4C-seq	Island-4	*del(1-10)*	Proximal Forelimb	1
4C-seq	Island-4	*del(8-13)rXII*	Distal Forelimb	2
4C-seq	Island-4	*del(8-13)rXII*	Proximal Forelimb	2
4C-seq	Island-4	*del(8-13)d11lac*	Distal Forelimb	1
4C-seq	Island-4	*del(8-13)d11lac*	Proximal Forelimb	1
4C-seq	Island-4	*del(1-13)d9lac*	Distal Forelimb	1
4C-seq	Island-4	*del(1-13)d9lac*	Proximal Forelimb	1
4C-seq	Island-4	*del(1-13)d11lac*	Distal Forelimb	2
4C-seq	Island-4	*del(1-13)d11lac*	Proximal Forelimb	2
4C-seq	Island-4	*del(attP-Rel5)d9lac*	Distal Forelimb	1
4C-seq	Island-4	*del(attP-Rel5)d9lac*	Proximal Forelimb	1
4C-seq	*Evx2*	*Wt*	Distal Forelimb	3
4C-seq	*Evx2*	*Wt*	Proximal Forelimb	3
4C-seq	*Evx2*	*del(10-12)*	Distal Forelimb	1
4C-seq	*Evx2*	*del(10-12)*	Proximal Forelimb	1
4C-seq	*Evx2*	*del(1-10)*	Distal Forelimb	1
4C-seq	*Evx2*	*del(1-10)*	Proximal Forelimb	1
4C-seq	*Evx2*	*del(8-13)rXII*	Distal Forelimb	1
4C-seq	*Evx2*	*del(8-13)rXII*	Proximal Forelimb	1
4C-seq	*Evx2*	*del(1-13)d11lac*	Distal Forelimb	1
4C-seq	*Evx2*	*del(1-13)d11lac*	Proximal Forelimb	1
4C-seq	*Hoxd13*	*Wt*	Distal Forelimb	3
4C-seq	*Hoxd13*	*Wt*	Proximal Forelimb	3
4C-seq	*Hoxd13*	*del(10-12)*	Distal Forelimb	1
4C-seq	*Hoxd13*	*del(10-12)*	Proximal Forelimb	1
4C-seq	*Hoxd13*	*del(9-12)*	Distal Forelimb	1
4C-seq	*Hoxd13*	*del(9-12)*	Proximal Forelimb	1
4C-seq	*Hoxd13*	*del(1-10)*	Distal Forelimb	1
4C-seq	*Hoxd13*	*del(1-10)*	Proximal Forelimb	1
4C-seq	*Hoxd4*	*Wt*	Distal Forelimb	3
4C-seq	*Hoxd4*	*Wt*	Proximal Forelimb	3
4C-seq	*Hoxd4*	*del(9-12)*	Distal Forelimb	1
4C-seq	*Hoxd4*	*del(9-12)*	Proximal Forelimb	1
4C-seq	*Hoxd4*	*del(8-13)d11lac*	Distal Forelimb	1
4C-seq	*Hoxd4*	*del(8-13)d11lac*	Proximal Forelimb	1
4C-seq	*Hoxd4*	*del(8-13)rXII*	Distal Forelimb	2
4C-seq	*Hoxd4*	*del(8-13)rXII*	Proximal Forelimb	2
4C-seq	CS38	*WT*	Distal Forelimb	5
4C-seq	CS38	*WT*	Proximal Forelimb	5
4C-seq	CS38	*del(10-12)*	Distal Forelimb	1
4C-seq	CS38	*del(10-12)*	Proximal Forelimb	1
4C-seq	CS38	*del(9-12)*	Distal Forelimb	1
4C-seq	CS38	*del(9-12)*	Proximal Forelimb	1
4C-seq	CS38	*del(1-10)*	Distal Forelimb	1
4C-seq	CS38	*del(1-10)*	Proximal Forelimb	1
4C-seq	CS38	*del(8-13)rXII*	Distal Forelimb	2
4C-seq	CS38	*del(8-13)rXII*	Proximal Forelimb	2
4C-seq	CS38	*del(8-13)d11lac*	Distal Forelimb	1
4C-seq	CS38	*del(8-13)d11lac*	Proximal Forelimb	1
4C-seq	CS38	*del(1-13)d9lac*	Distal Forelimb	1
4C-seq	CS38	*del(1-13)d9lac*	Proximal Forelimb	1
4C-seq	CS38	*del(1-13)d11lac*	Distal Forelimb	1
4C-seq	CS38	*del(1-13)d11lac*	Proximal Forelimb	1
4C-seq	CS38	*del(attP-Rel5)d9lac*	Distal Forelimb	1
4C-seq	CS38	*del(attP-Rel5)d9lac*	Proximal Forelimb	1
4C-seq	CS65	*Wt*	Distal Forelimb	1
4C-seq	CS65	*Wt*	Proximal Forelimb	1
4C-seq	lacZ	*del(attP-Rel5)d9lac*	Distal Forelimb	1
4C-seq	lacZ	*del(attP-Rel5)d9lac*	Proximal Forelimb	1
4C-seq	CS65	*del(attP-Rel5)d9lac*	Distal Forelimb	1
4C-seq	CS65	*del(attP-Rel5)d9lac*	Proximal Forelimb	1

Supplemental Table S2. Distribution of replicates used for 4C-seq experiments. Number of 4C-seq experimental replicates done for each set of viewpoints and tissue.

Rodriguez-Carballo307769_Table S3

Figure	Genotype	Tissue	Method	Antibody	Library name	Comments	Machine	# reads	# reads after cutadapt	Mapping rate	Duplication rate (from macs2)	Predicted Fragment length (from macs2)	#tags after filtering for the pile up (from macs2)
2A	Wt	Distal Forelimb	ChIP	H3K27ac	DFL_E12_Wt_H3K27ac		Hi-Seq 2500	201'198'677	198'705'462	94%	27%	202	135'636'166
2A	Wt	Distal Forelimb	ChIP	RAD21	DFL_E12_Wt_RAD21_rep1_10m		Hi-Seq 4000	25'523'795	25'233'395	98%			
2A	Wt	Distal Forelimb	ChIP	RAD21	DFL_E12_Wt_RAD21_rep2_25m	(biological replicate)	Hi-Seq 4000	22'778'021	22'624'958	98%			
2A	Wt	Distal Forelimb	ChIP	RAD21	merge						19%	180	37'906'210
2A	Wt	Proximal Forelimb	ChIP	H3K27ac	PFL_E12_Wt_H3K27ac		Hi-Seq 2500	212'060'763	209'489'871	92%	35%	207	126'285'072
2A,2B	Wt	Distal Forelimb	ChIP	CTCF	DFL_E12_Wt_CTCF		Hi-Seq 2500	181'140'348	179'502'879	88%	53%	212	74'288'903
2A,2B	Wt	Distal Forelimb	ChIP	Smc1	DFL_E12_Wt_SMC1_rep1_25m		Hi-Seq 2500	47'737'339	47'287'579	96%			
2A,2B	Wt	Distal Forelimb	ChIP	Smc1	DFL_E12_Wt_SMC1_rep2_25m	(biological replicate)	Hi-Seq 4000	24'360'674	20'338'202	98%			
2A,2B	Wt	Distal Forelimb	ChIP	Smc1	merge						21%	150	51'337'221
2A,2B,S2B	Wt	Proximal Forelimb	ChIP	CTCF	PFL_E12_Wt_CTCF		Hi-Seq 2500	189'433'084	187'826'261	95%	54%	220	81'348'527
S2A,S2B	del(1-13)d9lac	Whole Limb	ChIP	CTCF	Limbs_E12_del1-13d9lac_CTCF		Hi-Seq 4000	29'491'536	26'015'766	91%	63%	189	14'834'792
S2A	del(8-13)d11lac	Whole Limb	ChIP	CTCF	Limbs_E12_del8-13d11lac_CTCF		Hi-Seq 4000	41'621'892	41'142'783	94%	26%	224	28'751'023
S2A	del(8-13)rXII	Whole Forelimb	ChIPmentation	CTCF	Limbs_E12_del8-13rXII_CTCF	ChIPmentation	Hi-Seq 2500	22'965'580	22'759'718	99%	14%	97	19'318'942
S2A,S2B	Wt	Whole Limb	ChIP	CTCF	Limbs_E12_Wt_CTCF		Hi-Seq 4000	45'645'591	44'515'903	88%	66%	186	21'246'603
S2B	del(attP-Rel5)d9lac	Whole Limb	ChIP	CTCF	Limbs_E12_delattP-Rel5d9lac_CTCF		Hi-Seq 2500	47'091'011	46'223'083	93%	55%	185	19'421'727
S2C	del(8-13)rXII	Whole Limb	ChIP	RAD21	Limbs_E12_del8-13rXII_RAD21_seq1		Hi-Seq 2500	26'225'995	25'888'649	76%			
S2C	del(8-13)rXII	Whole Limb	ChIP	RAD21	Limbs_E12_del8-13rXII_RAD21_seq2	(resequencing of the library)	Hi-Seq 4000	52'448'941	52'309'855	78%			
S2C	del(8-13)rXII	Whole Limb	ChIP	RAD21	merge						20%	180	48'330'898
S2C	Wt	Whole Limb	ChIP	RAD21	Limbs_E12_Wt_RAD21		Hi-Seq 4000	317'343'381	314'560'310	98%	29%	197	218'522'862

Supplemental Table S3. Information on ChIP experiments. List of the different ChIP data-sets generated on this study, which includes methodological information as well as number of sequencing reads.

Supplemental Figures S1 to S7

Rodriguez-Carballo307769_FigS1

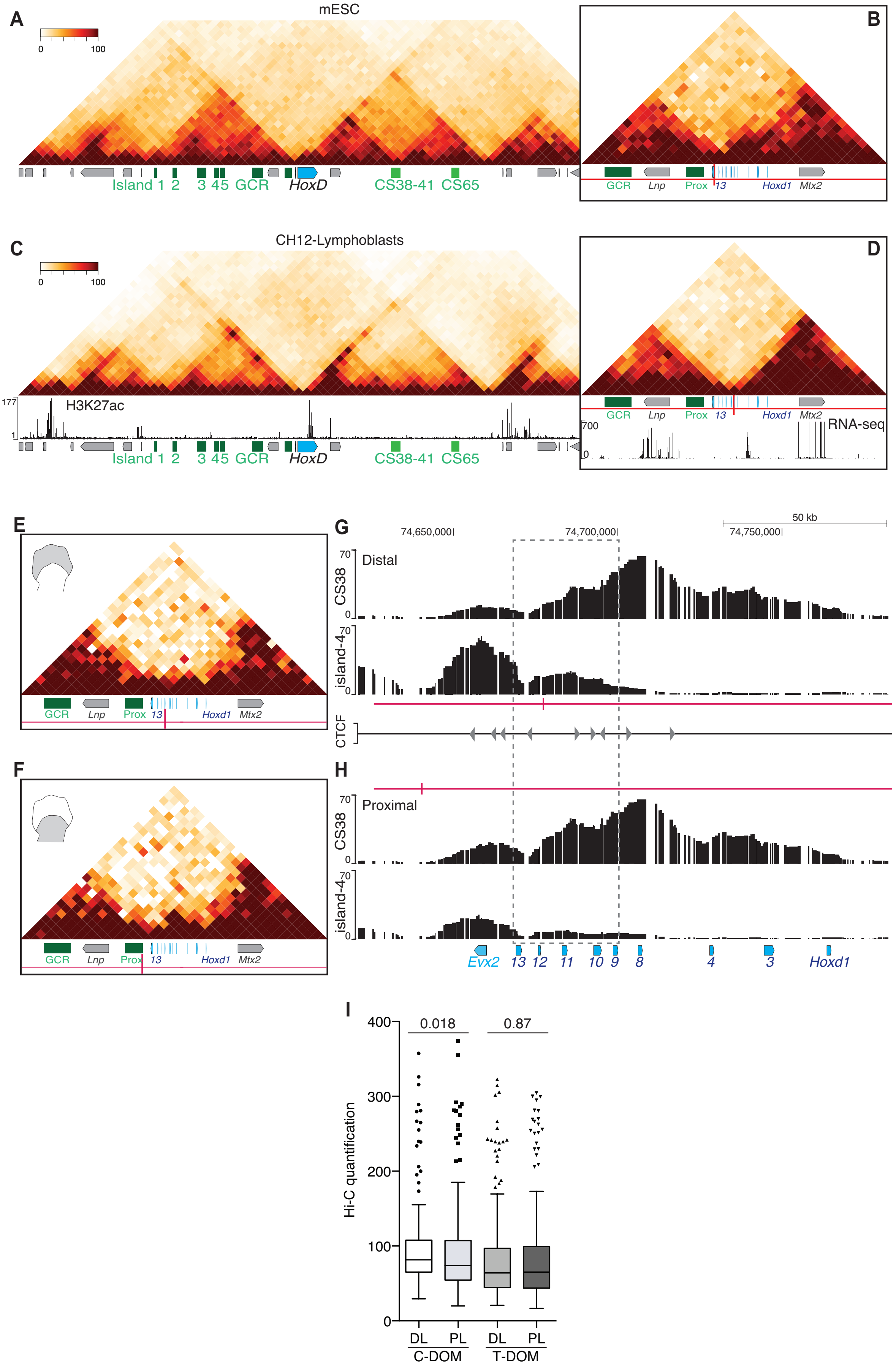

Supplemental Figure S1. Interactions between CS38 and island-4 with the *HoxD* cluster. **(A)** Hi-C heatmap at 40 kb resolution of mESC re-analysed and re-mapped on the mm10 genome (dataset from (Dixon et al. 2012)). **(B)**. Magnification of panel **(A)**, binned at a 20 kb resolution and using TopDom for boundary identification (red bar). **(C)**. *In-situ* Hi-C of CH12 lymphoblasts at a 40 kb resolution, re-analyzed and re-mapped on the mm10 genome from (Rao et al. 2014). Below the Hi-C heatmap is shown the ENCODE ChIP-seq data of H3K27ac in the same cells. **(D)**. Magnification of panel **(C)** binned at a 20 kb resolution and using TopDom for boundary identification (red bar). Below panel D is shown the ENCODE RNA-seq data of the same cells. **(E)**. Hi-C heatmap in distal limb cells at the *HoxD* locus, binned at a 20 kb resolution. The red line below indicates the position of the TAD boundary identified by using TopDom on the 40 kb matrix. (**F**)**.** Hi-C profile in proximal limb cells at the *HoxD* locus binned at a 20 kb resolution. The red line below indicates the position of the TAD boundary identified by using TopDom on the 40 kb matrix. **(G-H)**. 4C-seq contacts between CS38 and island-4 with *HoxD* in distal (**G**) and proximal (**H**) cells. Below panels G and H are shown *Hoxd* genes and the orientations of the various bound CTCF sites. The dotted line demarcates the limits of the dynamic *HoxD* boundary. The red bars between the tracks position the TAD boundaries as identified by the TopDom algorithm on limb-cells Hi-C. (**I**). Hi-C quantification of interactions on C-DOM and T-DOM of distal limb (DL) and proximal limb (PL) datasets. *p*-values for the Wilcoxon rank sum test between distal and proximal cells are shown on top of the graphs.

Rodriguez-Carballo307769_FigS2

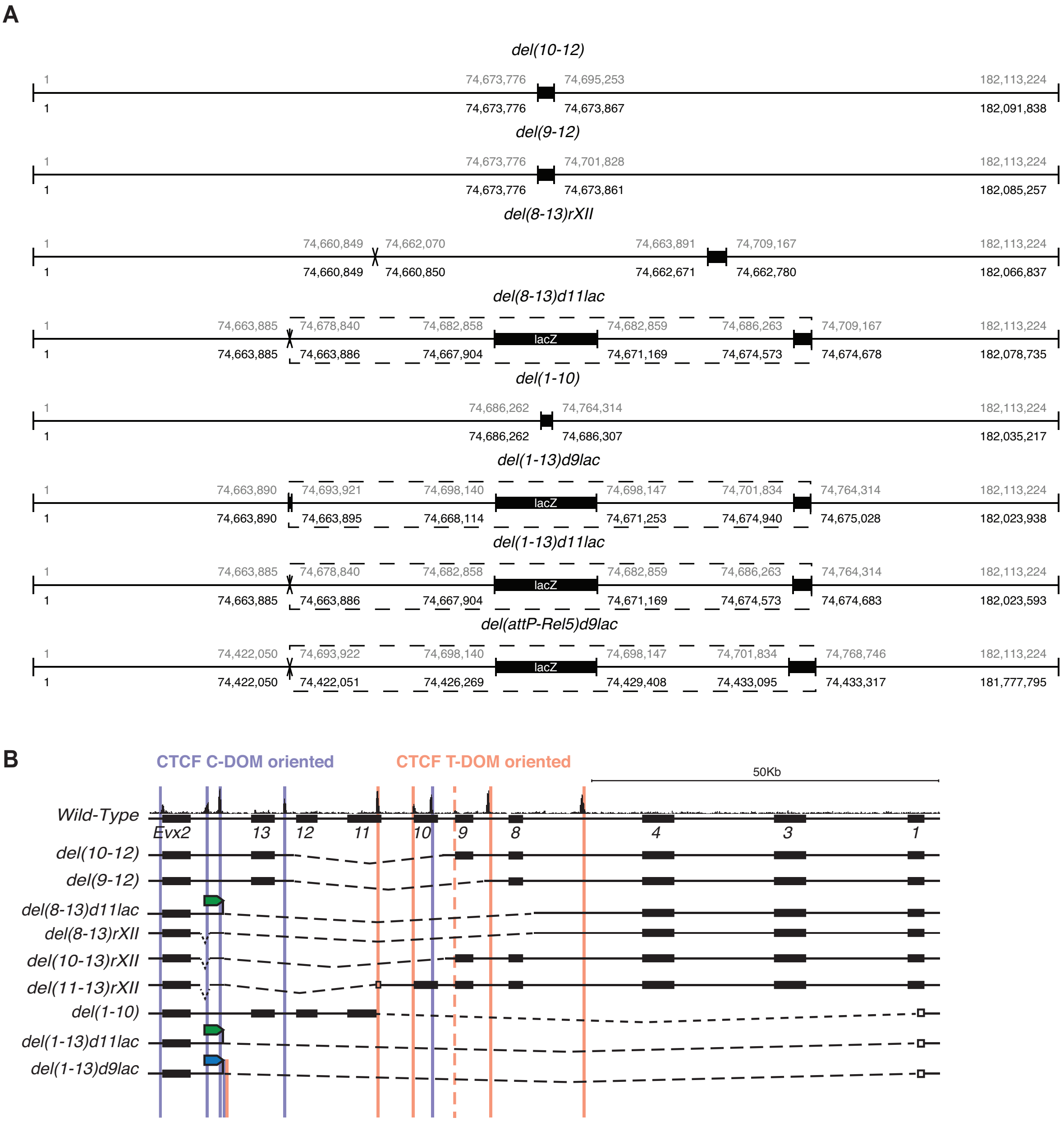

Supplemental Figure S2. **Schematics of the various mutant mouse lines. (A)**. Schemes of the various *in silico* constructed mutant genomes. The schemes indicate the various correspondences between the wild-type coordinates (shown on top of each configuration, above, in grey font) and the newly assigned coordinates for each mutant configuration (below, black font). The black boxes represent the *lacZ* transgene when present (lacZ), and the newly placed sequences associated either with *loxP* sites (for the *HoxD*$^{del(10\text{-}12)}$, *HoxD*$^{del(9\text{-}12)}$, *HoxD*$^{del(8\text{-}13)rXII}$, *HoxD*$^{del(8\text{-}13)d11lac}$, *HoxD*$^{del(1\text{-}10)}$, *HoxD*$^{del(1\text{-}13)d9lac}$ and the *HoxD*$^{del(1\text{-}13)d11lac}$ alleles) or with the remains of homology arms for the *HoxD*$^{del(attP\text{-}Rel5)d9lac}$ allele. **(B)**. List of the different mouse lines used in this study. The red lines indicate the CTCF sites orientated

towards the T-DOM, whereas blue lines indicate CTCF sites pointing towards C-DOM. The *Hoxd11lacZ* transgene does not contain any bound CTCF. The *Hoxd9lacZ* transgene contains one CTCF oriented towards C-DOM and a cryptic CTCF (shown as a red dashed line) facing the T-DOM. Region XII (rXII) removes the second C-DOM CTCF. The insertion of the *lacZ* transgenes at the *Nsi* site (5' of *Hoxd13*) does not affect the presence of bound CTCF at the nearby-located sites.

Rodriguez-Carballo307769_FigS3

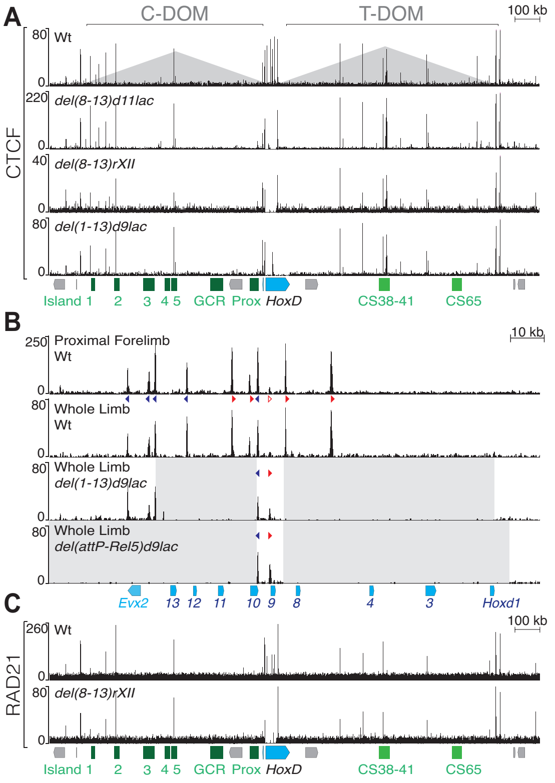

Supplemental Figure S3. Distribution of bound CTCF and RAD21 in limb cells at the wild type and mutant *HoxD* loci. (A). CTCF ChIP profiles of E12.5 limb cells in several mutant lines in which various parts of the *HoxD* cluster are deleted. The extents of the C-DOM and T-DOM TADs are shown as grey pyramids on top. The mutant alleles are shown on the left. **(B)**. CTCF ChIP-seq profiles of proximal forelimb and whole limb control, *HoxD*$^{del(1\text{-}13)d9lac}$ and *HoxD*$^{del(attP\text{-}Rel5)d9lac}$. The arrowheads above tracks indicate the orientation of the CTCF motifs inside the *HoxD* cluster. The empty arrowhead indicates the position and orientation of the cryptic CTCF that appears evident on the *Hoxd9lac* transgene. Grey rectangles indicate the extension of the deletions in *HoxD*$^{del(1\text{-}13)d9lac}$ and *HoxD*$^{del(attP\text{-}Rel5)d9lac}$. **(C).** RAD21 ChIP-seq profiles of E12.5 in wild type and *HoxD*$^{del(8\text{-}13)rXII}$ mutant limb cells. Below, the *HoxD* cluster is shown (blue) as well as regulatory sequences (green).

Rodriguez-Carballo307769_FigS4

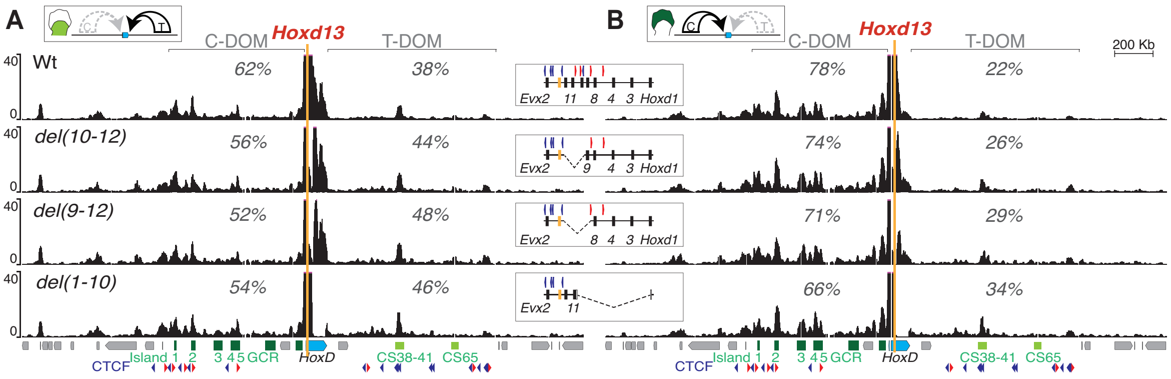

Supplemental Figure S4. Interaction profiles are modified upon partial removal of *Hoxd* genes. (A-B). 4C-seq using *Hoxd13* as a viewpoint in proximal (**A**) and distal (**B**) limb cells of either wt, *HoxD*$^{del(10\text{-}12)}$, *HoxD*$^{del(9\text{-}12)}$ or *HoxD*$^{del(1\text{-}10)}$. Schemes of the deletion alleles are shown on the centre. Below are shown the *HoxD* cluster (blue) and the various regulatory elements (green). The positions of C-DOM and T-DOM are indicated on top and their relative quantification is shown for each 4C-track.

Rodriguez-Carballo307769_FigS5

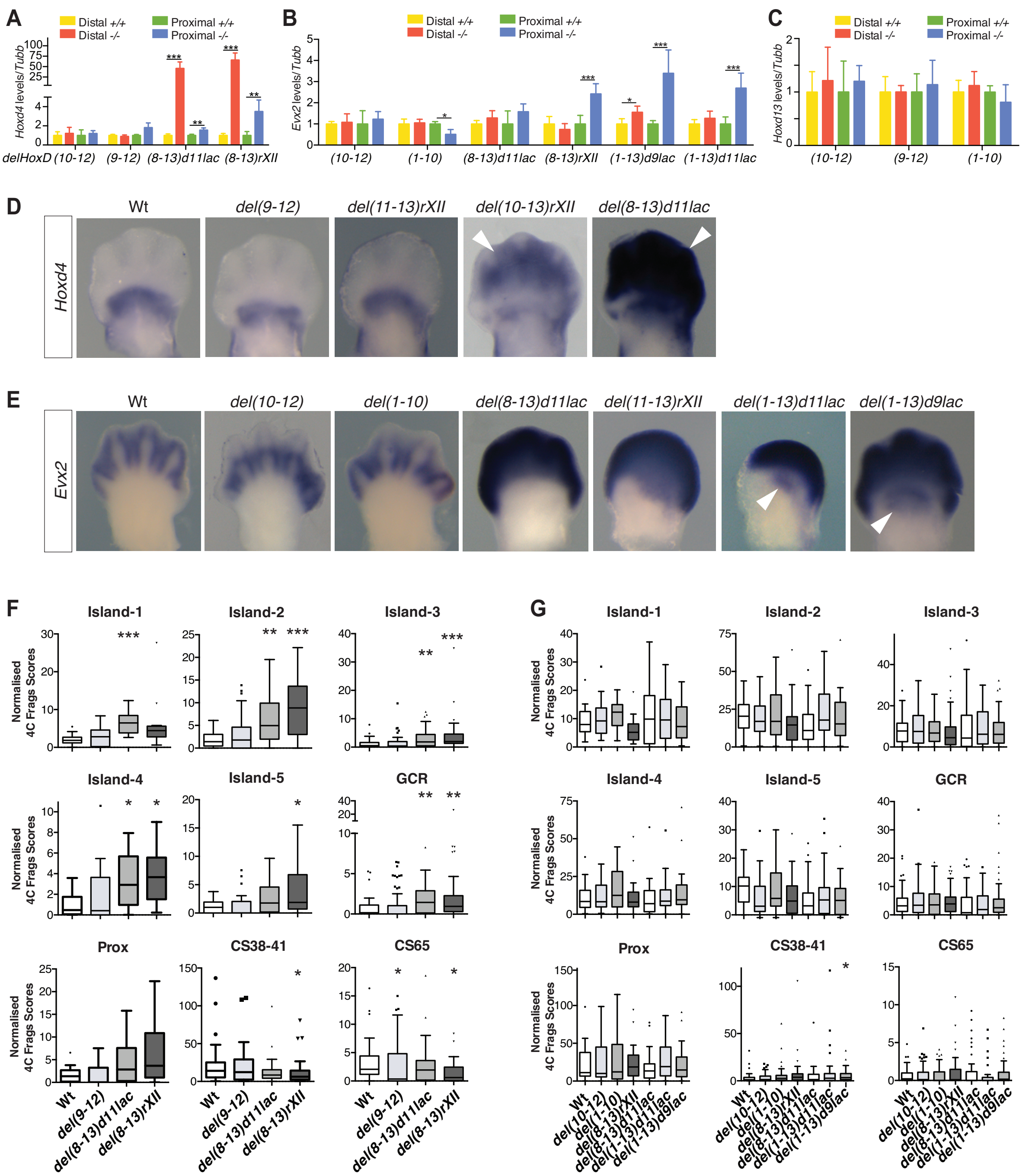

Supplemental Figure S5. Ectopic expression correlates with gained interactions. (A-C). RT-qPCR measures of *Hoxd4, Evx2* and *Hoxd13* mRNA steady state levels in several deletion alleles, either in distal or in proximal limb cells at E12.5. t-test corrected by Holm-Sidak Method. *p<0.05; **p<0.01; ***p<0.001. The -/- symbol means cells homozygous for the deletion indicated below. (**D-E**). WISH of E12.5 limbs dissected from several deletion mutants, stained either with a *Hoxd4* (**D**) or an *Evx2* (**E**) specific probe. The gain of

expression of *Hoxd4* and *Evx2* in distal and proximal cells, respectively, are indicated by arrowheads. (**F-G**). Normalized 4C fragments scores for *Hoxd4* (**F**) and *Evx2* (**G**) at several specific regions. Quantifications were performed from data obtained by using E12.5 distal and proximal limb cells, respectively. Kruskal-Wallis test **$p<0.01$; ***$p<0.001$.

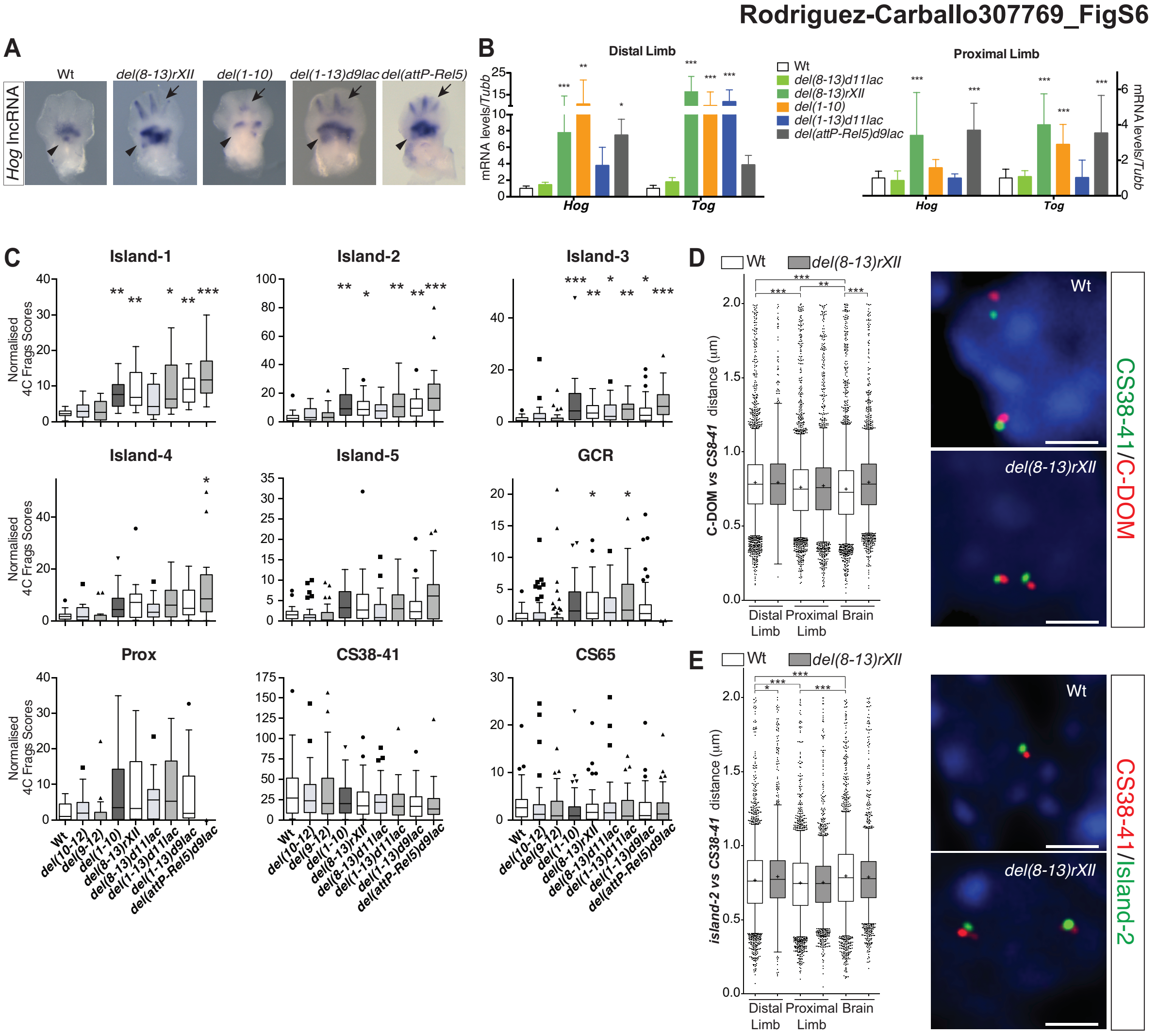

Supplemental Figure S6. Interaction profiles are modified upon partial removal of *Hoxd* genes. (**A**). WISH of the *Hog* lncRNA in wt, *HoxD*$^{del(8\text{-}13)rXII}$, *HoxD*$^{del(1\text{-}10)}$, *HoxD*$^{del(1\text{-}13)d9lac}$ and *HoxD*$^{del(attP\text{-}Rel5)d9lac}$ mutant alleles. The proximal domain is shown with arrowheads and the ectopic distal expression with arrows. (**B**). RT-qPCR values of *Hog* and *Tog* lncRNA mRNAs in various deletion alleles in distal (left) and proximal (right) limb bud cells at E12.5. (**C**). Interactions expressed as normalized 4C fragment scores between CS38 used as a viewpoint (see Fig. 5A) and the various sequences indicated at the top. Statistical analysis of mutant *versus* wt values performed using a Kruskal-Wallis test: *p<0.05; **p<0.01; ***p<0.001. (**D**). Representative images (scale: 2µm) and distance quantification of DNA-FISH visualising a C-DOM BAC *versus* a CS38-41 fosmid clone, either in wt (distal n=4718; proximal n=4406; brain n=3845) or in *HoxD*$^{del(8\text{-}13)rXII}$ limb bud and forebrain cells

(distal n=3015; proximal n=3395; brain n=3203). Fosmid and BAC positions are shown in Fig. 1B. Statistical analysis performed using a Kruskal-Wallis test: **p<0.01; ***p<0.001. (**E**). Representative images (scale: 2µm) and distance quantification of DNA-FISH visualising a CS38-41 *versus* an island-2 fosmid clones, either in wt (distal n=3559; proximal n=3575; brain n=3062) or in $HoxD^{del(8\text{-}13)rXII}$ limb bud and forebrain cells (distal n=2319; proximal n=2803; brain n=2649). Fosmid positions (dark green box for island-2 and pink box for CS38-41) are shown in Fig. 1B. Statistical analysis performed using a Kruskal-Wallis test: *p<0.05; **p<0.01; ***p<0.001.

Rodriguez-Carballo307769_FigS7

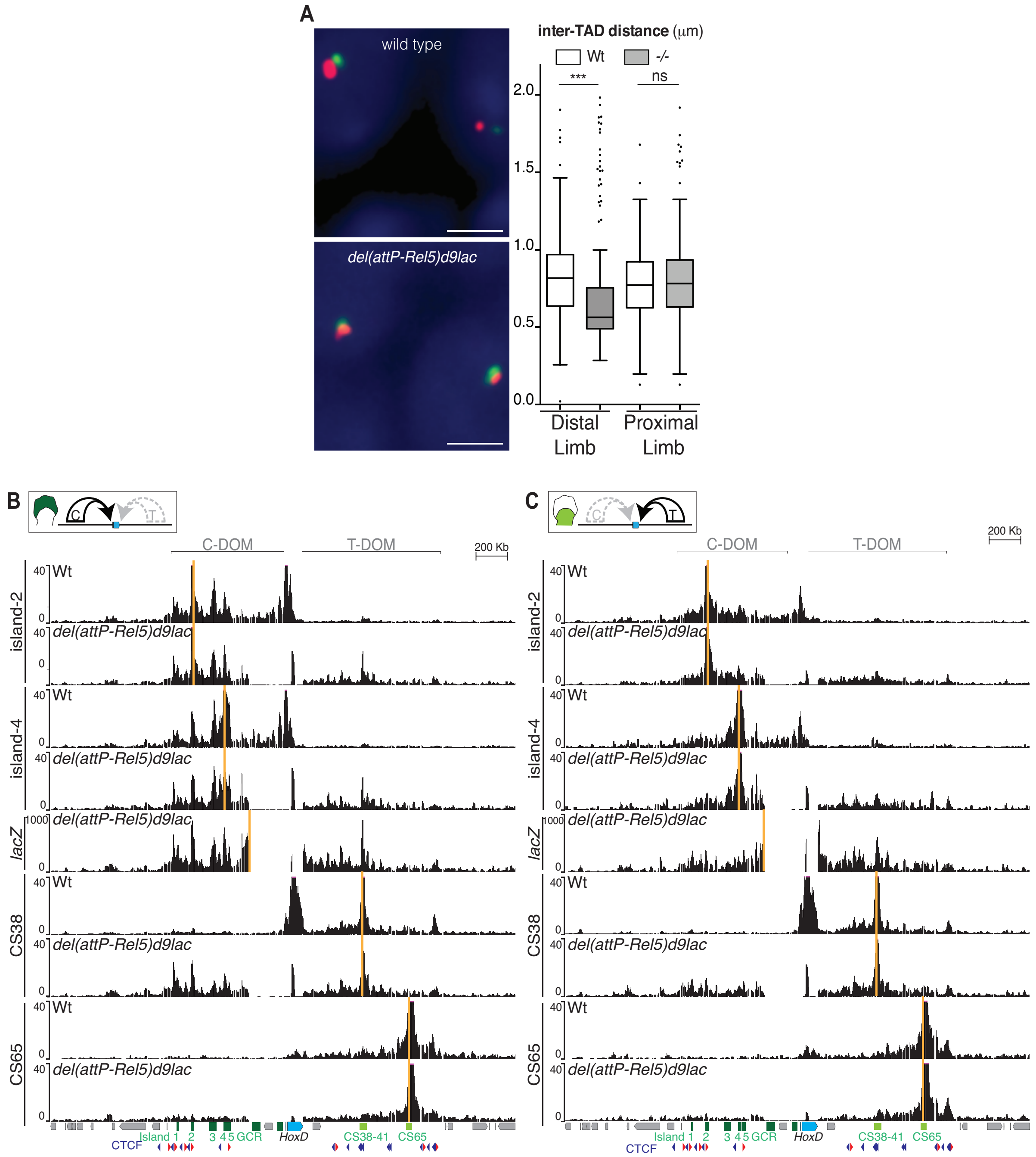

Supplemental Figure S7. High-resolution analysis of interaction profiles in the *HoxD*$^{del(attP\text{-}Rel5)d9lac}$ mutant allele. (**A**). Representative images (left) and inter-probe quantification (right) of DNA-FISH of control (distal n=387; proximal n=363) and *HoxD*$^{del(attP\text{-}Rel5)d9lac}$ (distal n=125; proximal n=378) mutant alleles using BAC clones covering C-DOM and T-DOM (scale: 2μm). Statistical analysis was performed using a Kruskal-Wallis test, with ***$p<0.001$. **(B-C)**. 4C-seq profiles of distal (**B**) and proximal (**C**) limb bud cells at E12.5 in the *HoxD*$^{del(attP\text{-}Rel5)d9lac}$ mutant stock. Island-2, island-4, *lacZ*, CS38

and CS65 were used as viewpoints, as indicated on the left. The *HoxD* cluster is indicated below (blue) as well as regulatory sequences (green). The positions of the respective viewpoints are shown with an orange vertical line. The positions of the C-DOM and T-DOM are shown on top.

REFERENCES

Dixon JR, Selvaraj S, Yue F, Kim A, Li Y, Shen Y, Hu M, Liu JS, Ren B. 2012. Topological domains in mammalian genomes identified by analysis of chromatin interactions. *Nature* **485**: 376–80.

Rao SS, Huntley MH, Durand NC, Stamenova EK, Bochkov ID, Robinson JT, Sanborn AL, Machol I, Omer AD, Lander ES, et al. 2014. A 3D map of the human genome at kilobase resolution reveals principles of chromatin looping. *Cell* **159**: 1665–80.
